# Supplementary material for: Closed magnetic topology in the Venusian magnetotail and ion escape at Venus
Source: Nat Commun. 2024 Jul 18;15:6065. doi: 10.1038/s41467-024-50480-0 (PMC11258336; doi:10.1038/s41467-024-50480-0)
Supplement: Supplementary file 1 — Supplementary Information [file 41467_2024_50480_MOESM1_ESM.pdf]

Supplementary Information For "Closed Magnetic Topology in the Venusian Magnetotail and Ion Escape at Venus"

Shaosui Xu[1*], David L. Mitchell[1], Phyllis Whittlesey[1], Ali Rahmati[1], Roberto Livi[1], Davin Larson[1], Janet G. Luhmann[1], Jasper S. Halekas[2], Takuya Hara[1], James P. McFadden[1], Marc Pulupa[1], Stuart D. Bale[1,3], Shannon M. Curry[1,4] and Moa Persson[5]

[1]Space Sciences Laboratory, University of California Berkeley, 7 Gauss Way, Berkeley, 94720, California, USA.
[2]Department of Physics and Astronomy, University of Iowa, Iowa City, Iowa, USA.
[3]Physics Department, University of California Berkeley, 366 Physics North MC 7300, Berkeley, 94720, California, USA.
[4]Department of Astrophysical and Planetary Sciences, University of Colorado Boulder, 2000 Colorado Ave, Boulder, 80305, Colorado, USA.
[5]Swedish Institute of Space Physics, Uppsala, Sweden.

*Corresponding author E-mail:** shaosui.xu@ssl.berkeley.edu

Contributing authors: mitchell@ssl.berkeley.edu; phyllisw@berkeley.edu; rahmati@ssl.berkeley.edu; rlivi@berkeley.edu; davin@berkeley.edu; jgluhman@ssl.berkeley.edu; jasper-halekas@uiowa.edu; hara@ssl.berkeley.edu; mcfadden@ssl.berkeley.edu; pulupa@berkeley.edu; bale@berkeley.edu; shannon.curry@colorado.edu; moa.persson@irf.se

Contents of the file:

Supplementary figures 1-4.

Supplementary Note:

In the Supplementary Information, we use data from the Pioneer Venus Orbiter (PVO) mission and the Venus Express (VEx) mission. Both the PVO and VEx data are obtained from the publicly available database AMDA (https://amda.irap.omp.eu/). The VEx electron pitch angle distribution data products are publicly available at NASA Planetary Data System (https://doi.org/10.17189/m6w1-6q39).

Supplementary Discussion:

Supplementary Figure 1 displays the distributions of magnetic field strength at Venus at altitudes <200 km (altitudes of footpoints of open/closed topology) measured by the magnetometer onboard the Pioneer Venus Orbiter (PVO) spacecraft from 1979 to 1988. The PVO data are obtained from the publicly available database AMDA (https://amda.irap.omp.eu/). It shows that the magnetic field at the Venus nightside collisional atmosphere is greater than 20 nT at ~ 40% of the time. This is in good agreement with our estimated magnetic strength at the footpoint of open/closed field lines.

Supplementary Figure 2 displays the zoomed-out view of the measured magnetic fields by PSP during its 4th Venus flyby. It shows that the upstream IMF was quite variable before and after Venus's magnetosphere's crossing.

Supplementary Figure 3 shows the minimum variance analysis (MVA) over the period of closed field lines, which gives inconclusive results regarding the exact configuration of the flux rope, as the PSP did not cut through the center of the possible flux rope. The fitting results are: the maximum (B_i), intermediate (B_j), and minimum (B_k) variance directions in the VSO frame are as follows: B_i = [0.051, 0.954, 0.297], B_j = [-0.880, 0.183, -0.438], B_k = [-0.472, -0.239, 0.849]. The eigenvalues for each direction are λ_i = 13.339, λ_j = 1.211, and λ_k = 0.286, respectively. The eigenvalue ratio between intermediate and minimum variance direction (λ_j / λ_k) is computed to be ~4.2.

Supplementary Figure 4 shows an example of Venus Express's (VEx) plasma and magnetic field observations [1] crossing the magnetotail (time interval indicated by the black bar in the second panel). The VEx data are obtained from the publicly available database AMDA (https://amda.irap.omp.eu/) and the electron pitch angle distribution data products are publicly available at NASA Planetary Data System: https://doi.org/10.17189/m6w1-6q39. In this case study, the tail magnetic topology is mainly draped. Meanwhile, both the protons and heavy ions are mostly hot and/or energetic (ram ion energies from the spacecraft velocity <1 eV). This case study presents an example of hot/energetic ions associated with the draped topology (field lines not connected to the Venus ionosphere).

Supplementary Figures:

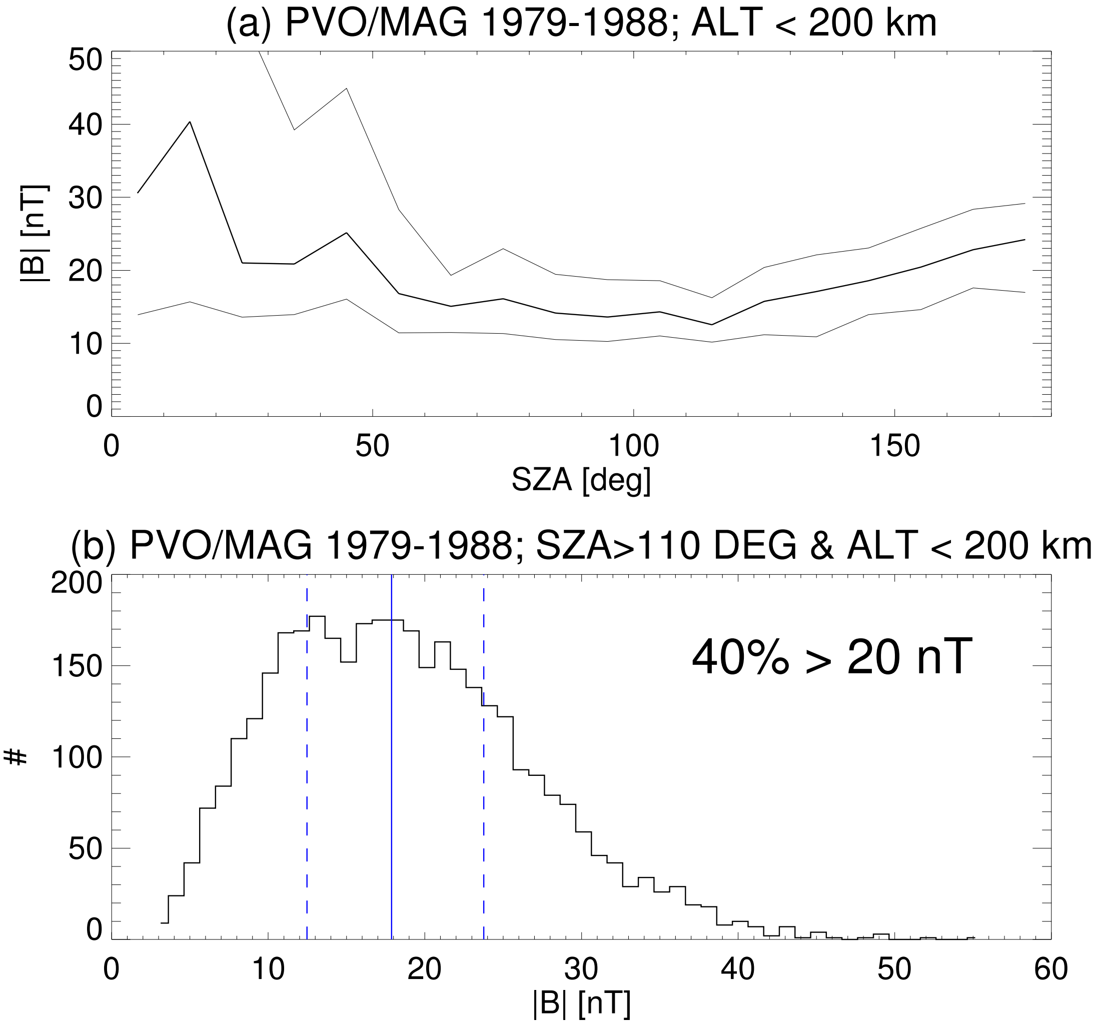

Supplementary Figure 1. Results of magnetic field amplitudes measured by the magnetometer onboard the Pioneer Venus Orbiter (PVO) spacecraft from 1979 to 1988, limited to altitudes below 200 km (altitudes of footpoints of open/closed topology). Data are obtained from the publicly available database AMDA (https://amda.irap.omp.eu/). (a) The lines are the quartiles of the magnetic field strength as a function of solar zenith angle (SZA). (b) The distribution of magnetic field strength for SZA> 110° (nightside), with the three vertical lines showing the quartiles. As labeled in panel b, the measured magnetic field strength on the nightside (SZA> 110°) at low altitudes (<200 km) is greater than 20 nT at ~ 40% of the time.

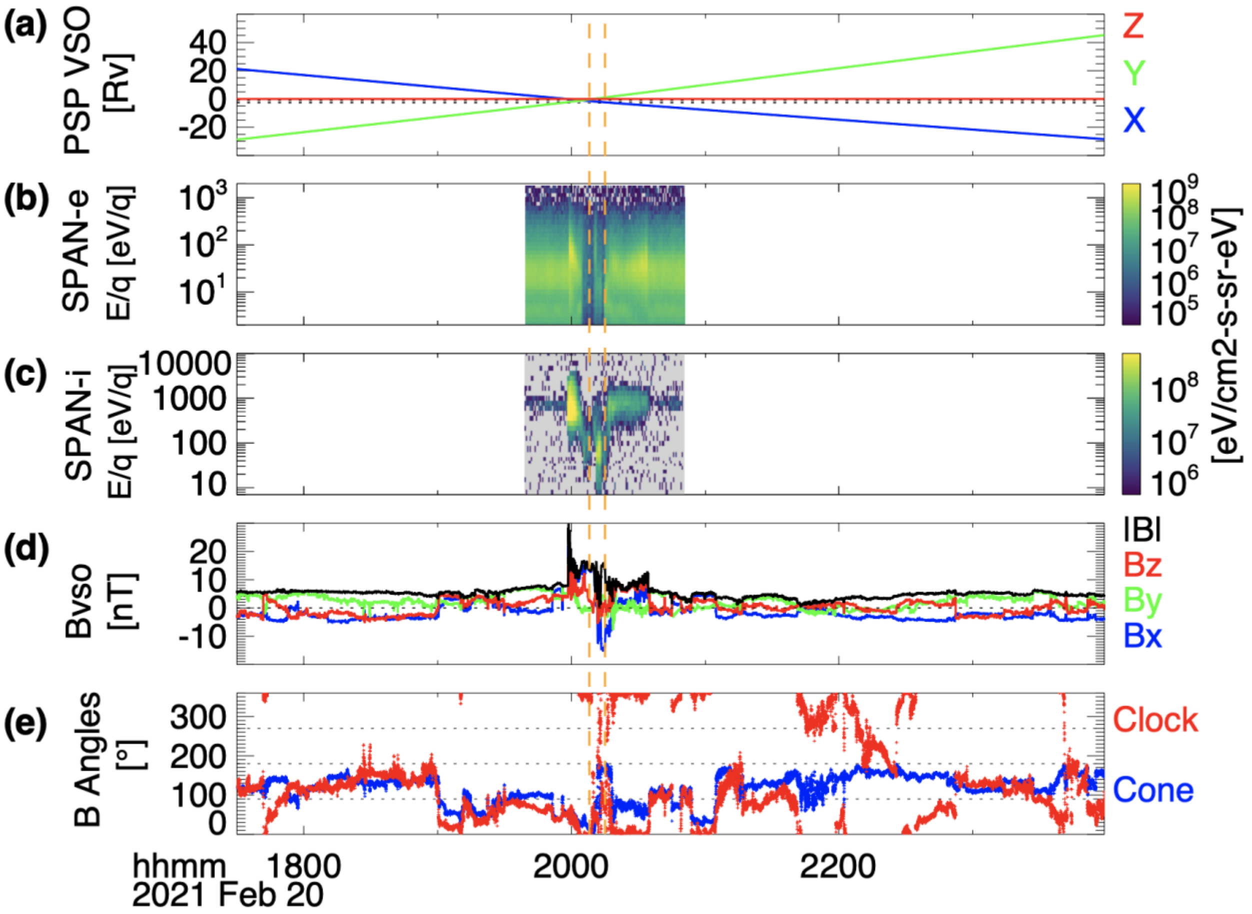

Supplementary Figure 2. Time series of the PSP observations on 20 February 2021 during its 4th Venus gravity assist: (a) the PSP trajectory in the Venus-Solar-Orbital (VSO) coordinates, (b) omnidirectional electron differential energy fluxes measured by the SPAN-e instrument, (c) ion energy differential energy fluxes averaged over all look directions measured by the SPAN- i instrument, (d) the 1-s magnetic field vector and strength and (e) magnetic clock angles and cone angles in the VSO coordinates, measured by the FIELDS instrument.

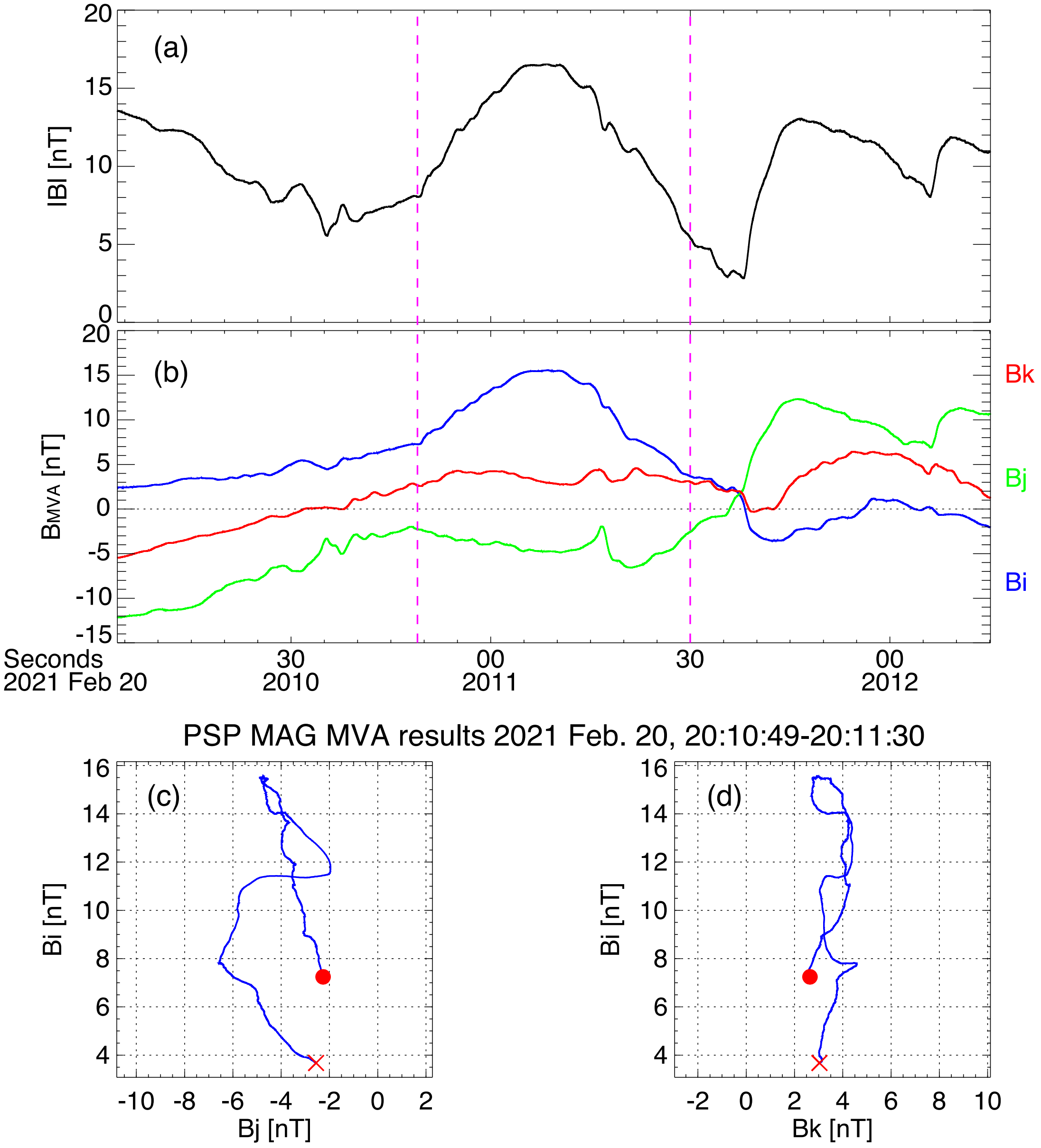

Supplementary Figure 3. (a-b) Time series of magnetic field strengths (a) and vectors (b) in the VSO coordinates measured by the PSP/FIELD instrument. (c-d). Results of minimum variance analysis (MVA) over the time interval marked by the two dashed lines in panels a-b, (c) Bi vs Bj, and (d) Bi vs Bk, where Bi, Bj, Bk are the maximum, intermediate, and minimum variance directions.

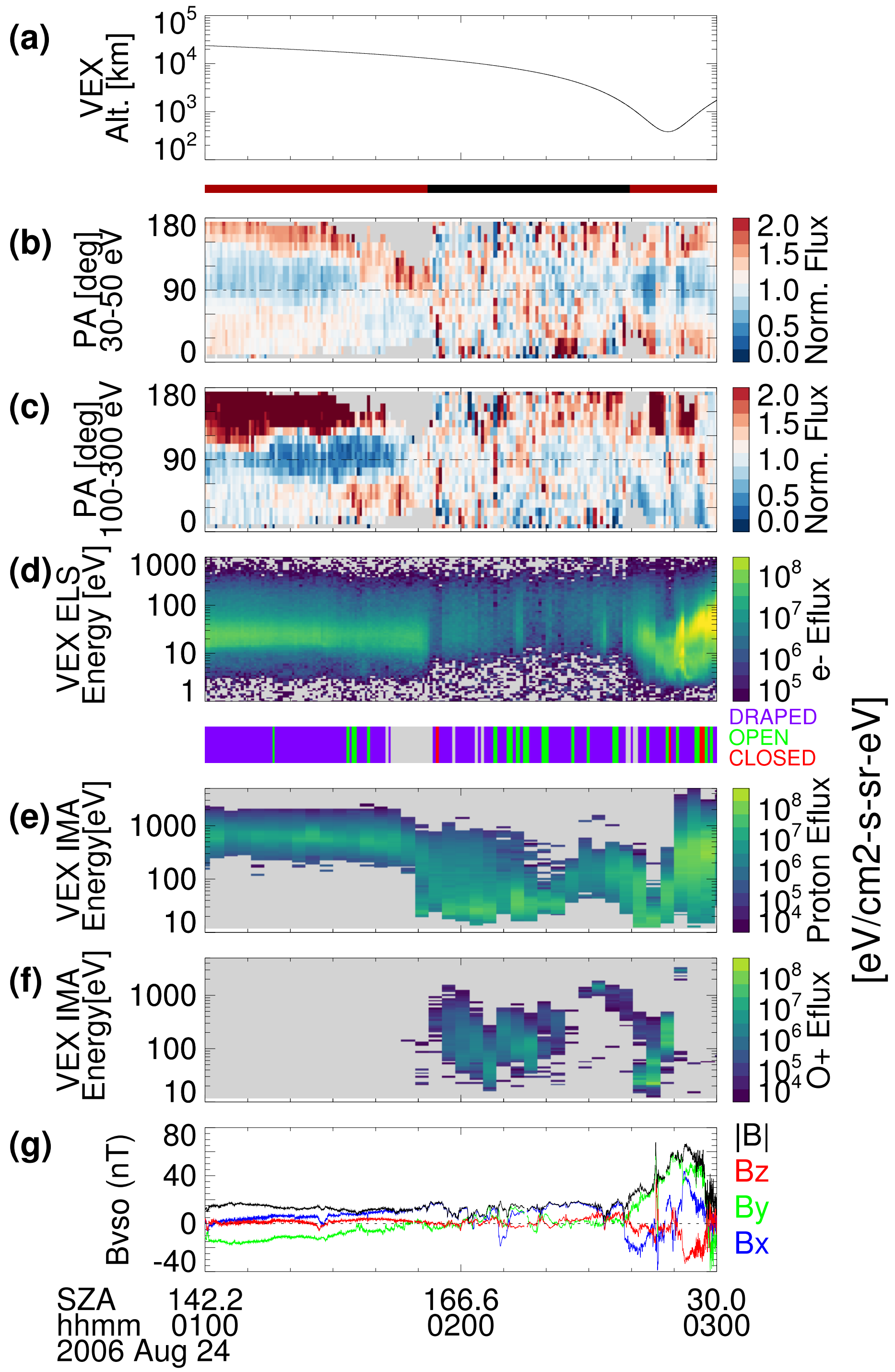

Supplementary Figure 4. Time series of VEx (Venus Express) observations on 24 August 2006: (a) VEx's altitude, a color bar for sunlit (red) and darkness (black), (b) electron pitch angle distribution (PAD) of 30-50 eV, (c) electron PAD of 100-300 eV, and (d) omnidirectional electron energy spectra, magnetic topology derived from automatic procedures provided by Xu et al., [2023][2], (e) heavy and (f) light ion energy spectra averaged over all look directions, and (g) the magnetic field vector and strength in the VSO coordinates.

Supplementary References:

[1] Barabash, S., Sauvaud, J.-A., Gunell, H., Andersson, H., Grigoriev, A., Brinkfeldt, K., Holmstrom, M., Lundin, R., Yamauchi, M., Asamura, K., et al.: The analyser of space plasmas and energetic atoms (ASPERA-4) for the Venus Express mission. Planetary and Space Science 55(12), 1772–1792 (2007)

[2] Xu, S., Frahm, R.A., Ma, Y., Luhmann, J.G., Mitchell, D.L., Persson, M.: Statistical mapping of magnetic topology at venus. Journal of Geophysical Research: Space Physics 128(12), 2023–032133 (2023). https://doi.org/10. 1029/2023JA032133
